# Identifying Antimicrobial Agents from *Chlorella sorokiniana*: A Biotechnological Approach Utilizing Eco-Friendly Extraction and Characterization Methods

**DOI:** 10.3390/biotech14010022

**Published:** 2025-03-18

**Authors:** Elia Lio, Martina Dramis, Gianluca Ottolina, Francesco Secundo

**Affiliations:** 1 Department of Pharmaceutical Sciences, University of Milan, Via Mangiagalli 25, 20133 Milan, Italy; elia.lio@unimi.it; 2 Istituto di Scienze e Tecnologie Chimiche “Giulio Natta”, Consiglio Nazionale delle Ricerche, Via Mario Bianco 9, 20131 Milan, Italy; m.dramis@campus.unimib.it (M.D.); gianluca.ottolina@scitec.cnr.it (G.O.)

**Keywords:** green organic solvent, antimicrobial, bioactive compounds, microalgae, PCA, GC-MS

## Abstract

Natural compounds are increasingly favored over synthetic ones for their lower environmental impact. However, extraction and characterization processes typically rely on harsh conditions and conventional solvents, which are unsustainable and cause pollution. This study aimed to develop an eco-friendly extraction method to isolate and evaluate the antimicrobial properties of bioactive compounds from *Chlorella sorokiniana*. Using dimethyl carbonate (DMC), methoxycyclopentane (CPME), and butan-2-one (MEK) as green solvents alongside chloroform as a non-green reference solvent, on both untreated and sodium hydroxide pre-treated microalgae biomass, extract yields of up to 182 ± 27 mg/g DW were obtained using MEK. Extracts from untreated microalgae biomass exhibited lower MIC values compared to those obtained with the same solvent from pre-treated biomass, when tested as antimicrobial agents against *Escherichia coli*, *Bacillus megaterium*, and *Bacillus subtilis*. The lowest MIC value (4.89 ± 0.05 µg/mL) was observed against *E. coli* using the extract from the untreated microalgae biomass with CPME, which was comparable to the vancomycin control (1.55 ± 0.03 µg/mL). Principal component analysis highlighted correlations between GC-MS-identified compounds and antimicrobial activity. ANOVA and post hoc tests (*p* < 0.05) confirmed solvent choice, and pre-treatment influenced yield and bioactivity. The results underscore green solvents as sustainable alternatives for extracting bioactive compounds from autotrophic microalgae.

## 1. Introduction

Bioactive compounds isolated from photosynthetic microorganisms, such as microalgae, exhibit numerous chemical and functional properties, which have been the driving force behind the development of microalgae and cyanobacteria large-scale cultivations [1]. Microalgae can grow in autotrophic conditions, producing biomass that is useful in various industrial and pharmaceutical applications. In addition to primary metabolites, microalgae are rich in various bioactive compounds, including carotenoids, polyphenols, and polyunsaturated fatty acids [2,3]. They are a natural source of bioactive molecules, including compounds of pharmaceutical interest [4]. Microalgae are increasingly studied for their potential in antibiotic, antiviral, and anticancer drug discovery [4,5,6,7]. In addition, the isolable bioactive compounds have significant potential applications in veterinary medicine and agriculture [3].

Within this context, *C. sorokiniana* emerges as an ideal model organism for the study and extraction of bioactive compounds. This green microalga is widely cultivated due to its resilience to variable environmental conditions and its rapid growth, even in autotrophic settings, and it is recognized for its ability to produce a wide array of secondary metabolites, which have shown biological activity in multiple studies [8].

A key challenge in harnessing microalgal potential is developing efficient extraction methods and characterizing their pharmaceutical properties (e.g., antimicrobial activity). Methods such as supercritical fluid extraction, pressurized liquid extraction, ultrasound-assisted extraction, and microwave-assisted extraction can reduce environmental impact. While these methods provide a more sustainable alternative to conventional organic solvents, challenges remain in their large-scale application, particularly in achieving comparable extraction yields. Industrial processes have traditionally used solvents like chloroform and hexane, which, despite environmental concerns, often offer superior extraction efficiency and selectivity for specific bioactive compounds. Therefore, optimizing green extraction methods is essential to improve yield without compromising compound stability or purity, emphasizing the need for further comparative studies and process refinements [9].

Building on these considerations, green solvents have emerged as promising environmentally friendly alternatives to conventional solvents [10,11,12,13]. In the present study, dimethyl carbonate (DMC), methoxycyclopentane (CPME), and butan-2-one (MEK) were chosen as model green solvents due to their suitability for the tested extraction processes [14,15,16].

The composition of extracts from natural sources often remains unidentified, as extraction, purification, and isolation are time- and resource-intensive processes that significantly impact the environment, particularly when solvents or harsh conditions are involved [17]. A viable alternative is GC-MS analysis, which can detect a wide range of compounds, including lipids, terpenes, and microalgae-specific metabolites. Thus, it provides at least a partial insight into the chemical composition of the extracts [18]. In addition, applying principal component analysis (PCA) to GC-MS data can reveal correlations between identified molecules and their specific effects (e.g., antimicrobial activity), eliminating the need for further purification, at least during preliminary screening [19].

This study aims to develop sustainable, eco-friendly extraction methods for microalgal biomass by optimizing the extraction and identification of antimicrobial compounds while minimizing environmental impact and streamlining analytical processes. In addition, we seek to establish a correlation between the chemical composition of these extracts and their antimicrobial activity, thereby highlighting the industrial potential of *C. sorokiniana* microalgae as valuable sources of bioactive compounds for food preservation and bacterial infection management.

## 2. Materials and Methods

### 2.1. Microalgae Strain Identification and Growth Conditions

A non-characterized microalgae strain named JB3 was donated by Prof. Pengcheng Fu laboratory, at Beijing University of Chemical Technology, Beijing, China, and it was subjected to strain identification. DNA was extracted from freeze-dried biomass using a commercial column-based kit and quantified with the Qubit™ 4.0 Fluorometer (Invitrogen™, Thermo Fisher Scientific, Waltham, MA, USA). PCR was performed using ITS1f and ITS4r primers, and the amplification was verified via 1% agarose gel electrophoresis with the E-GEL™ Power Snap System (Invitrogen™). Sanger sequencing followed a standard protocol involving ExoSAP purification, BigDye sequencing reaction, and analysis on the 3500 Genetic Analyzer (Applied Biosystems™, Thermo Fisher Scientific, Waltham, MA, USA). The Sanger sequencing yielded DNA fragments of 723 base pairs (bp). These sequences were subjected to basic local alignment search tool (BLAST 2.16.0+) against the NCBI nucleotide database. The alignment results indicated that the sequenced fragments showed 100% sequence identity to *Chlorella sorokiniana*. The identified strain was differentiated from other *C. sorokiniana* strains adding the suffix JB3.

*C. sorokiniana* JB3 was cultivated using minimal culture media, specifically the BG-11 medium [20], preparing a pre-inoculum culture of 100 mL volume from a single colony grown on Petri dishes with 2% agar in BG-11. When cell growth reached an optical density (OD_680_) at 680 nm of 0.8 Abs (measured by a V-730 UV-Vis Spectrophotometer (Jasco, Tokyo, Japan)), the pre-inoculum was added to 4 L autoclaved culture media in a 5 L bottle and air fluxed (flow rate 10 mL/min) to favor culture agitation. Air was sterilized by a filter (ReZist PTFE-S Filter, 50 mm/0.2 µm (Whatman™, Cytiva, Marlborough, MA, USA). The biomass was harvested at the stationary phase OD_680_ ≈ 1.46 after eleven days. Illumination was provided by a bank of photosynthetic lamps (67702172-C Photosynthetic Lamps C-LED Srl, Imola, Italy), with an intensity of 24.05 µmol/m^2^/s, an emission spectrum of 360–760 nm, and emission peaks at 450 nm and 680 nm, respecting the circadian cycle of the microalgae with 14 h of light and 10 h of darkness. Cultivation temperature was maintained at 25 ± 1 °C. The growth of *C. sorokiniana* was conducted in triplicate to ensure reproducibility and statistical reliability of the data.

Aliquots were withdrawn at 24 h intervals from the start of cultivation (t_0_) to monitor the growth through OD_680_ measure. Once the cells reached the stationary phase, the biomass was harvested through centrifugation for 30 min at 4500 RCF and 4 °C. The wet biomass was subsequently frozen at −80 °C, lyophilized, and then stored at −20 °C.

### 2.2. Preparation of Extracts

For the extraction process, biomass was suspended in the desired organic solvent: deionized H_2_O (80:20 *v*/*v*) mixture. Then, it was placed on ice and subjected to 3 cycles (2 min each) of sonication using an Omni Ruptor 250-Watt Ultrasonic Cell Disruptor (Cole-Parmer Instrument Company, Vernon Hills, IL, USA), operating at 50% of the maximum sonication power. The green solvents employed in this study were dimethyl carbonate (DMC) [21], methoxycyclopentane (CPME) [22], and butan-2-one (MEK) [23], while chloroform (CLF) was used as a non-green reference solvent. The role of water in the mixture was essential to facilitate effective cell rupture and enable the formation of a biphasic system [24]. Following sonication, the organic phase was collected, and the solvent was subsequently removed using a rotary evaporator at a temperature of 30 °C. The same procedure was applied to the control samples without microalgae [11]. All extractions and chlorophyll removal process were performed in triplicate to ensure reproducibility and accuracy of results.

When the biomass was pre-treated for chlorophyll (Chl) removal, the biomass was preventively bleached with sodium hydroxide. Chl from freeze-dried microalgae biomass was removed as described in [25]. A total of 14 mL of solution obtained by mixing a 1% (*w*/*w*) NaOH solution with ethanol (1:4, *v*/*v*) was added to 100 mg of the lyophilized biomass and heated to 70 °C for 1 h. Subsequently, the ethanol was evaporated using a rotary evaporator, and the desired green organic solvent was added to the residual aqueous phase to obtain the extracted solution. The Chl removal yield was determined spectrophotometrically using the following equation [26]:

Chlorophyll removal yield (%) = [(A_0_ − A_1_)/A_0_] × 100, (1)

where A_0_ and A_1_ represent the absorbance at 680 nm of the extracted solution before and after removal, by recording the spectra from 750 to 500 nm.

The E factor was calculated as reported by Sheldon [27].

### 2.3. Antimicrobial Tests of the Extracts

The agar well diffusion method and broth dilution method were used to assess the antimicrobial activity of the extracts against *Escherichia coli* (ATCC BAA-1025-B1) (American Type Culture Collection, Manassas, VA, USA), *Bacillus megaterium* (ATCC 14581) (American Type Culture Collection, Manassas, VA, USA), and *Bacillus subtilis* (ATCC 6051) (American Type Culture Collection, Manassas, VA, USA).

#### 2.3.1. Agar Well Diffusion Method

The agar well diffusion method involved inoculation of the entire surface of Mueller–Hinton agar plates with a microbial culture with an optical density (OD 600 nm) of 0.2 [28]. Aseptically, wells with a diameter of 7 ± 1 mm were created in the agar using a sterile cork borer or tip. Next, 50 µL of a 1 mg/mL solution of the extract dissolved in 300 µL 5% Tween-20 and 10% DMSO aqueous solution was dispensed into the wells, and the agar plates were then incubated at 25 °C for 24 h. A 1 mg/mL vancomycin solution was used as a positive control. The extract components or vancomycin diffusion into the agar medium formed a round halo where the microbial growth was inhibited.

#### 2.3.2. Broth Dilution Method

The broth dilution method involved preparing wells in a 96-well microtiter plate. In each well, 0.15 mL of a microbial inoculum (*E. coli*, *B. megaterium*, or *B. subtilis* adjusted to McFarland 0.5) was added [28]. Then, 0.15 mL of an extract prepared in an aqueous solution containing 5% DMSO and 10% Tween 20 was added to reach a final volume of 0.3 mL, yielding final extract concentrations of 0 (control), 12.5, 25, 62.5, 125, 250, or 500 µg/mL.

Each extract, prepared in triplicate, was tested in quintuplicate to ensure the reproducibility of the results. Subsequently, the plates were incubated for 24 h (25 °C, 100 rpm). Optical density was measured at 600 nm, and the minimum inhibitory concentration (MIC) was extrapolated according to the procedures outlined in the Supplementary Materials [29,30].

### 2.4. Extract Characterization—GC-MS Analysis

The extracts and their controls were analyzed by gas chromatography coupled to mass spectrometry (GC-MS ThermoQuest-Trace chromatograph GC—Finnigan-Trace DSQ MS), equipped with a column MEGA-1 MS 30 m × 0.25 mm × 0.25 µm, Crossbond (MEGA S.r.l, Legnano, Italy). The chromatographic analysis was conducted by heating from 60 °C (initial time 0.30 min) to 280 °C (heating rate 8 °C/min, final time 27.5 min) and 15 °C/min to 310 °C, with a split ratio of 50 and a column flow of 1.2 mL/min. The raw data derived from GC-MS chromatograms underwent several preprocessing stages, including normalization, following the subsequent formulas:

Differential chromatogram = Normalized chromatogram − Mean minus U-CLF,(2)

where:

Normalized chromatogram = Chromatogram intensities × (Total chromatogram intensities/∑Total chromatogram intensities),(3)

Total chromatograms mean = ∑of all chromatogram intensities/number of chromatograms Mean minus U-CLF = Total chromatogram mean − Normalized U-CLF(4)

### 2.5. Statistical Analysis and PCA

All analyses were conducted using R statistical software (v4.2.1; R Core Team 2022) [31]. Initially, one-way and two-way analysis of variance (ANOVA) were applied [32], followed by multiple comparison tests (Tukey and Duncan tests) to discern significant differences among the extracts [32,33]. The Tukey test was chosen due to its ability to control the Type I error rate across multiple comparisons, making it suitable for balanced designs with equal sample sizes. In cases where unequal variances were suspected, the Duncan test was applied as an alternative, being less conservative and more powerful in detecting differences, though with a higher risk of Type I errors. Principal component analysis (PCA) was performed using the singular value decomposition (SVD) algorithm [34]. Raw data from GC-MS chromatograms were normalized before performing PCA.

## 3. Results and Discussion

### 3.1. Microalgae Biomass Production

The chemical composition of microalgae extracts can vary not only according to the microalgae species but also due to the growing conditions (e.g., mixotrophic or autotrophic) [35,36].

**Figure 1:**
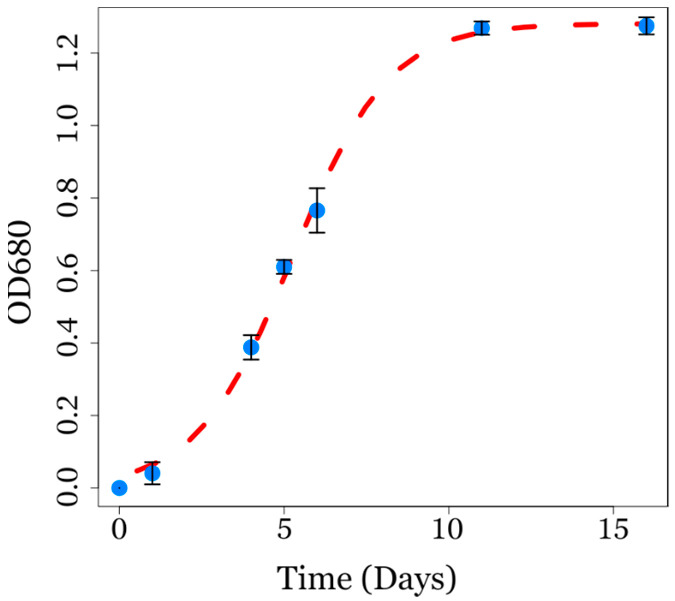
Growth curve of *Chlorella sorokiniana* cultivated in an aerated batch culture in a 5 L bottle. Values are means ± SD, *n* = 3. The blue markers indicate the mean OD_680_ values at different time points, while the error bars represent the corresponding standard deviations. The red dashed line represents the fitted exponential growth model.

In the present study, *C. sorokiniana* followed a typical cell growth of autotrophic microorganisms with an exponential phase (from day 2 to day 10) and a stationary growth phase (OD_680_ ≈ 1.46) after eleven days (Figure 1). The biomass was harvested at the stationary phase to obtain a dry biomass of 2.5 ± 0.8 g. The microalgae growth rate had a value of approximately 0.69 OD_680_/day, that is, a daily biomass increment of 69%. This rate of increase is in the range of a typical microalgae growth rate in autotrophic aerated batch culture [37], indicating non-stressed conditions and the robust vitality of the population and providing useful parameters to characterize the initial microalgal biomass.

Previous studies have shown that biomass composition and bioactive compound production can be influenced by growth conditions, reinforcing the need for controlled cultivation parameters to ensure reproducibility and optimal extraction yields [38]. Therefore, the yield of extracts and antimicrobial activity obtained in this study can be related to the microalgae biomass obtained as described above.

### 3.2. Extract Preparations

The extraction strategy for microalgal biomass depends on the chemical characteristics of secondary metabolites [39]. By using green organic solvents for the extraction process and starting from the microalgae biomass, we aimed to develop an environmentally sustainable procedure, diminishing resource consumption, mitigating the environmental impact, and making it more feasible for industrial applications [40,41].

However, besides secondary metabolites, microalgae biomass contains relatively high amounts of chlorophyll (Chl), which can affect the analytical characterization and antimicrobial assays of the extracts [25,26]. Because of this, two different extraction procedures were applied: (1) one from microalgae biomass previously pre-treated (e.g., NaOH saponification) to remove chlorophyll, and (2) one from untreated microalgae biomass, to compare the effects due to the presence of chlorophyll and simplify the extraction procedure. In both cases, the extraction was carried out with the same organic solvents, resulting in two groups of extracts: pre-treated (PT-) and untreated (U-) microalgae biomass.

#### 3.2.1. Extract Preparation from Pre-Treated Microalgae Biomass and Influence of the Solvents on Chlorophyll Removal

**Figure 2:**
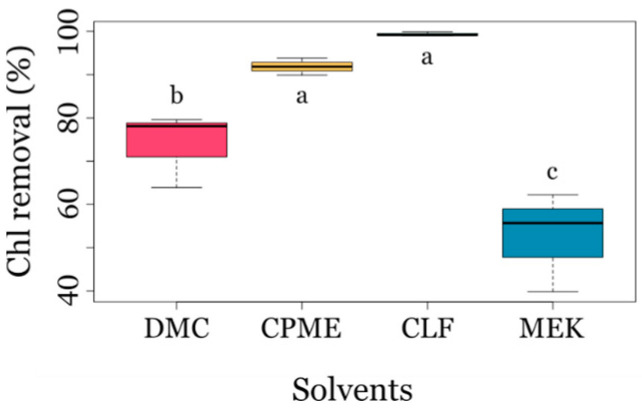
The boxplot illustrates the yields of chlorophyl removal after pre-treatment with sodium hydroxide, obtained with green organic solvents dimethyl carbonate (DMC), methoxycyclopentane (CPME), and butan-2-one (MEK) and non-green reference solvent chloroform (CLF). Following treatment, a diluted extract aliquot was employed to record UV-Vis spectra. Different letters (a, b, c) indicate statistically significant differences according to Duncan’s test.

Thanks to the pre-treatment of microalgae biomass, it was possible to reduce the amount of Chl in the extracts. However, the amount of Chl removed from the pre-treated microalgal biomass depended on the solvent used for extraction. CLF and CPME showed the highest Chl removal efficiencies (>92%). In contrast, those with more hydrophilic solvents, 73% for DMC and 52% for MEK, were significantly different from the removal obtained with CLF and CPME. This variation is likely due to the selective partitioning of Chl hydrolysis products, such as phytol and chlorophyllin, into the aqueous phase, which depends on the hydrophobicity of the organic solvent used (Figure 2). One-way ANOVA (Table S1), the post-hoc analysis Tukey test (Figure S1), and Duncan test revealed the significant impact of solvent type on Chl removal from microalgae extracts.

**Figure 3:**
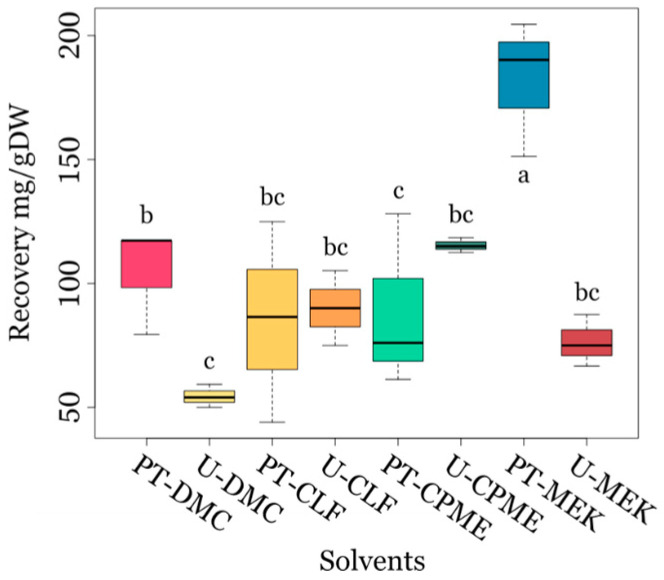
Boxplot of the extraction yields from biomass with (PT-) and without sodium hydroxide (U-) pre-treatment, using various organic solvents for extraction. Small lettering is shown according to Duncan’s test. Different letters (a, b, c, bc) indicate statistically significant differences according to Duncan’s test.

Interestingly, the microalgae biomass pre-treatment did not significantly affect the extract yields from the untreated biomass with organic solvents CLF and CPME, and they were even higher with DMC and MEK (Figure 3).

#### 3.2.2. Extracts from Untreated Microalgae Biomass

Extraction yields are depicted in the boxplots in Figure 3. A comparison of the extraction results was supported using a two-way analysis of variance ANOVA (shown in Table S1) and Tukey test (Figure S2). This statistical analysis was conducted considering both factors, i.e., the solvent employed for extraction and the pre-treatment method applied to the biomass before extraction. The analyses obtained using the “solvent factor” only or both the “solvent and pre-treatment factor with interaction” produced statistically significant outcomes (*p* < 0.05), which indicated a substantial solvent effect on the extraction yield. There is not significant statistical variation (*p* > 0.05) of the yield when considering the “pre-treatment factor” alone. The pre-treatment caused an increase in the extraction yield, except for CLF (around 87 ± 3 mg/g DW, either for pre-treated or untreated biomass) and resulted in the highest yield with MEK (182 ± 27 mg/g DW). In all other cases, yields were less than 88 ± 35 mg/g DW for the untreated samples and less than 115 ± 3 mg/g DW for the pre-treated samples. Duncan’s post-hoc analysis was performed to categorize solvents based on their impact on extraction yields (Figure 3).

These results align with findings from various research groups, which emphasize that optimizing extraction conditions, particularly solvent selection and pre-extraction treatments, is crucial for maximizing the yield of secondary metabolites and lipids from microalgae [42,43].

#### 3.2.3. Assessment of Extract Sustainability

To assess the sustainability of chemical extractions, the E factor [44] was calculated, as it measures the waste generated relative to the product obtained. This aids in optimizing processes, comparing options, complying with regulations, and promoting green chemistry. The extractions conducted using untreated biomass yielded lower E factor values compared to the treated samples extracted with the same solvent (Table 1). The increase of the E factor was attributed to the NaOH treatment for biomass saponification. With an ideal E factor of zero, chemical processes with E factors ranging from 5 to 50 kg waste/kg products can be considered green [27].

### 3.3. Antimicrobial Tests of the Extracts

To the best of our knowledge, only one study has investigated the antimicrobial effects of *C. sorokiniana* extracts [45]. In this study, the research group used only methanolic extracts and found that the extract of Chlorella sp. UKM8 exhibited the highest antibacterial activity without showing toxicity to Vero cells. Instead, the *C. sorokiniana* UKM2 extract did not demonstrate antimicrobial activity tested by the disc diffusion method. It is important to emphasize that using only one extraction method limits the identification of extracts with antimicrobial activity.

Based on these previous studies, we deemed it appropriate to evaluate the antimicrobial activity of different types of extracts obtained in the present work. Accordingly, untreated (U) and pre-treated (PT) microalgal biomass extracts, prepared using various organic solvents, were compared against *E. coli*, *Bacillus megaterium*, and *Bacillus subtilis*. These microorganisms were selected as model organisms based on information provided by the ATCC (https://www.atcc.org/, accessed on 1 February 2025)). In particular, *E. coli* is commonly used in media testing, quality control, water testing, and urinary tract infection research. *B. megaterium* serves as a quality control strain, while *B. subtilis* is employed for testing bacterial resistance to latex paint and ensuring sterility.

The antimicrobial activity of all the extracts was tested by (1) the agar well diffusion method and (2) the broth dilution method.

#### 3.3.1. Agar Well Diffusion Method

**Figure 4:**
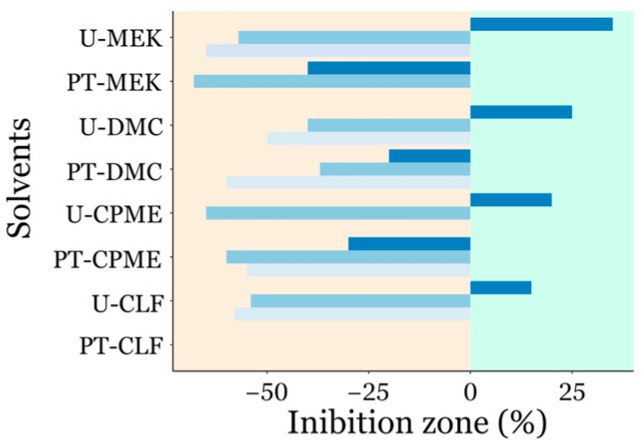
Inhibition zones of various extracts tested against *Escherichia coli* ■, *Bacillus megaterium* ■, and *Bacillus subtilis* ■. Inhibition zones are expressed as a percentage relative to vancomycin (positive control), normalized to its inhibition zone. Positive values indicate a greater effect, and negative values signify a weaker effect than vancomycin. The absence of the bar indicates no difference compared to the negative control (only 300 µL of an aqueous solution containing 5% Tween-20 and 10% DMSO).

Inhibition zones were expressed as a percentage relative to vancomycin (the positive control) (Figure 4). A key finding is that, in all cases, larger inhibition zones were observed with extracts obtained from the untreated microalgae biomass. Interestingly, against *E. coli*, the U-DMC, U-CPME, U-CLF, and especially U-MEK extracts exhibited inhibition zones exceeding those of vancomycin by +25%, +20%, +15%, and +35%, respectively. In all the other cases the extracts had reduced efficacy (up to –68% with PT-MEK) compared to vancomycin. In addition, PT-CLF and PT-MEK showed no antimicrobial activity with all microorganisms and with *B. subtilis*, respectively. No inhibition zone was observed with 5% Tween-20 and 10% DMSO aqueous solution (negative control).

These results indicate that microalgae biomass NaOH pre-treatment degrades bioactive compounds essential for antimicrobial activity toward all microorganisms. Chemical pre-treatments can break down thermolabile and pH-sensitive molecules [46]. The higher efficacy of extracts from untreated biomass may stem from preserving a broader metabolite spectrum.

The contrasting response against the Gram-positive *B. megaterium* and *B. subtilis* compared to the Gram-negative *E. coli* highlights that the extraction process favors the extraction of compounds with preferential activity against Gram-negative bacteria. Moreover, because Gram-negative bacteria generally exhibit higher resistance due to their outer membrane, the antimicrobial activity of the extracted metabolites might rise by their synergistic interactions, which enhances their activity against the integrity of bacterial cell wall structures [47,48]. However, it is worth pointing out that the variability in antimicrobial activity of the extracts observed by the well diffusion method stems from differences in physicochemical properties of active compounds (polarity, solubility, and molecular weight) that influence diffusion in agar media [49].

The different activity of the extracts obtained with different solvents from the same microalgae biomass (e.g., the most effective extract against *E. coli* was obtained using MEK) reflects the solvents’ polarity and their ability to extract distinct classes of bioactive compounds (e.g., fatty acids, phenols, peptides, terpenes) [50]. This observation may also explain the varying effects of *C. sorokiniana* extracts in the present study, as well as the lack of antimicrobial activity (or only modest activity against *E. coli*) reported by Shaima et al. [45], where only methanolic extracts were used.

#### 3.3.2. Broth Dilution Method

The comparison of the MIC values (Table 2) demonstrated that most extracts exhibited antimicrobial activity to some extent, confirming the trend observed in the inhibition zone test. Notably, the extracts showed greater effectiveness against the Gram-negative *E. coli*.

Controls with Tween-20 and DMSO did not affect cell viability. In all cases, the untreated extracts showed a 6-, 20-, and 16-fold higher MIC value than the pre-treated biomass, for *E. coli*, *B. megaterium*, and *B. subtilis*, respectively. In particular, the U-MEK and U-CPME extracts showed the lowest MIC values against all bacterial strains. The statistical analysis (Table S1) revealed significant impacts of ‘solvent’ (*p* > 0.03), ‘pre-treatment’ (*p* > 0.003), and ’bacteria’ factors on antimicrobial outcomes (*p*-values were >0.03, >0.003, and >0.04, respectively). Among the tested extracts, the U-CPME extract exhibited an MIC value (4.87 µg/mL) close to that of vancomycin (1.55 µg/mL) (Table 2), making it the most promising candidate for future applications. Its potential is further supported by the environmentally friendly procedure used for its production.

However, concerning the other extracts, it is important to emphasize that, in the context of industrial applications, antimicrobial activity should be viewed as the result of multiple bioactive compounds acting together rather than a single antibiotic-like effect. Although their antimicrobial activity may be lower than that of conventional antibiotics, this does not diminish their potential. Unlike antibiotics, which typically target specific bacterial mechanisms, natural extracts contain multiple bioactive compounds that can act synergistically. This multifaceted mode of action may reduce the risk of resistance development, making them valuable for long-term antimicrobial strategies. Therefore, while their direct efficacy may not match that of antibiotics, their broader mechanism and potential for sustainable application make them an important alternative for industrial and pharmaceutical use. There was a notable relation between “bacteria and solvents interaction factor” (*p* > 0.002), suggesting that the same solvent used for the extraction causes different antimicrobial strengths depending on the type of bacteria.

The biomass pre-treatment with sodium hydroxide caused a statistically significant reduction in the activity of the extracts [46]. Analogously, the “bacteria and pre-treatment interaction factor” was significant (*p* > 0.02) and had different effects on different bacterial strains. The reduction in antimicrobial activity following sodium hydroxide pre-treatment may be explained by the potential degradation or modification of essential bioactive compounds in the extracts. Sodium hydroxide can induce hydrolysis reactions, which may degrade or increase the negative charges in the compounds of the treated biomass, causing a lower affinity for the extracting solvents or even altering the molecular conformations needed for effective interaction with bacterial membranes, thereby reducing their antimicrobial efficacy [51].

### 3.4. Extract Characterization

#### 3.4.1. Principal Component Analysis (PCA) of GC-MS Chromatogram

**Figure 5:**
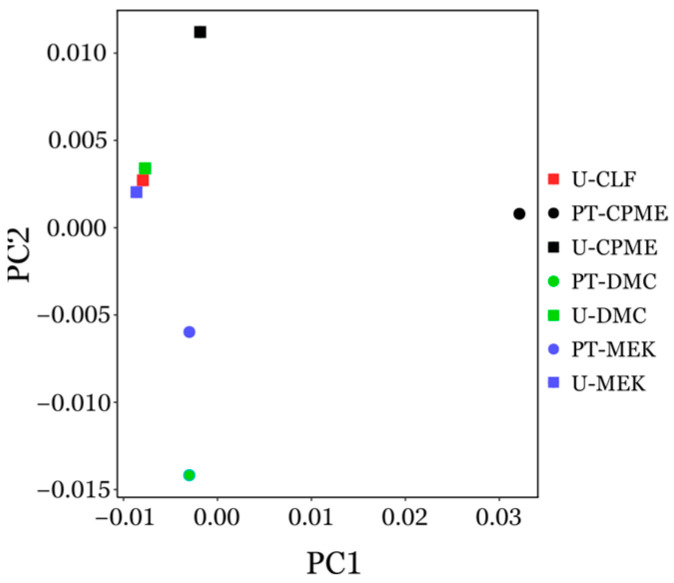
Principal component analysis of GC-MS chromatogram profiles of microalgae extracts.

The GC/MS profile for each extract showed more than 400 distinct peaks, revealing a high degree of complexity (Figure S3). To reduce this complexity, chromatograms were normalized and subjected to principal component analysis (PCA). The outcome of the PCA provided two eigenvectors, PC1 and PC2, which represent 62% and 19% of the total variance, respectively (Table S2). This suggests that the first two principal components captured most of the variation of the data. The PCA revealed two clusters with a clear distinction between pre-treated and untreated extracts (Figure 5), indicating that the pre-treatment causes a marked variation of the chemical composition of the extracts. In accordance with Table 2, untreated extracts not only have similar GC-MS chromatogram profiles, but they also show higher antimicrobial activity.

The compositional differences among the extracts were determined by generating differential chromatograms. This was achieved by subtracting the mean intensity of the eight chromatographic profiles from the profile of each individual extract (as detailed in Materials and Methods). Each extract yielded a distinct differential profile, and 15 peaks with a relative abundance greater than 0.001 were selected and assigned to their corresponding molecular structures (Table 3, Figure S4).

Interestingly, Shaima et al., using GC-MS analysis, found that the antimicrobial active extract of *Chlorella* sp. UKM8 contained 28 major compounds, including phenol, hexadecanoic acid, phytol, 9,12-octadecadienoic acid, and bicycloheptane, all known for their antimicrobial properties [45]. It is important to note that these authors did not report the molecular similarity scores, which might have led to the identification of an even greater number of compounds.

The molecules identified in our study include compounds (e.g., 2,6-ditert-butyl-4-methylphenol) that have been reported to exhibit antimicrobial activity and could theoretically contribute to the antimicrobial effects observed by Santhakumari et al., 2018 [52]. Conversely, other identified compounds, such as 2,6-ditert-butyl-4-hydroxy-4-methylcyclohexa-2,5-dien-1-one, bis(2-ethylhexyl) benzene-1,4-dicarboxylate, and 2,6-ditert-butyl-4-methylphenol, have not been previously reported as antimicrobial agents. However, their presence in extracts with demonstrated antimicrobial activity highlights them as potential antimicrobial candidates, warranting further investigation into their antimicrobial properties.

#### 3.4.2. Pearson Correlation

**Figure 6:**
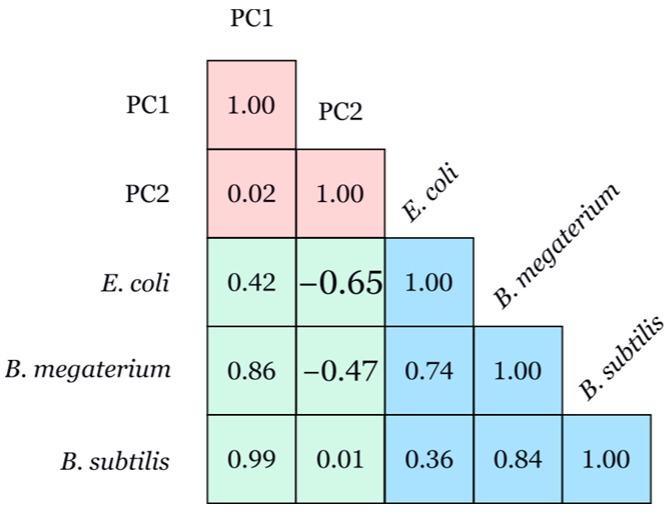
Correlogram of Pearson correlations between principal component and MIC values against *E. coli*, *B. megaterium*, and *B. subtilis*.

PC1 shows a positive correlation with *E. coli* (0.43), *B. megaterium* (0.86), and *B. subtilis* (0.99), indicating an increase in MIC for all three bacterial strains with increasing PC1 values (Figure 6). Consequently, extracts with higher PC1 values exhibit lower antimicrobial activity against these bacteria. PC2 exhibits a negative correlation with *E. coli* (−0.65) and *B. megaterium* (−0.48) and no correlation with *B. subtilis*. This suggests that an increase in PC2 values is associated with a decrease in minimum inhibitory concentrations and indicates greater antimicrobial activity against *E. coli* and *B. megaterium*.

## 4. Conclusions

A sustainable strategy for identifying antimicrobial compounds in microalgal biomass by employing cleaner methodologies was highlighted by (i) comparing extracts with and without the chlorophyll removal step; (ii) using green organic solvents as alternatives to chloroform; and (iii) integrating ANOVA, post hoc tests, and PCA on GC-MS chromatograms.

The findings underscored that, although the typical pre-treatment for chlorophyll removal is beneficial for analytical purposes, it may reduce antimicrobial potency, suggesting that leaving Chl intact can better preserve bioactivity in preliminary screenings. In addition, by avoiding the Chl removal step, the need for harsh and extensive purification processes is reduced, thereby streamlining and making the discovery process of antimicrobial agents from microalgae more sustainable.

Different extraction conditions and biomass treatment influence not only the extraction yield but also the antimicrobial efficacy of the extracts. However, the use of green solvents has proven to be a valuable alternative to CLF, whose toxic and polluting effects are more difficult to manage. The extract obtained with MEK from an untreated microalgal biomass was the most effective against *E. coli*. Therefore, future research exploring additional eco-friendly solvents could maximize extraction efficiency and minimize environmental impact.

Additionally, employing statistical analysis to correlate the molecules responsible for antimicrobial activity—identified within the complex mixture of compounds in an extract through GC-MS analysis—offers a sustainable approach for identifying potential candidate molecules for antimicrobial purposes.

Because the extracts were active against Gram-negative (*E. coli*) and Gram-positive bacteria (*B. subtilis* and *B. megaterium*), this approach may have broader applications in identifying antimicrobial molecules across various bacterial types. Testing a wider range of bacterial strains could better assess the broad-spectrum antimicrobial potential of microalgae-derived compounds, advancing their application in various industries.

## Figures and Tables

**Table 1:** E factors for extracts obtained from treated or untreated dry *Chlorella sorokiniana* biomass.

Extraction Methods	E-Factor (mg Waste/mg Extract) ^1^
PT-DMC	21.9
U-DMC	17.4
PT-CPME	28.7
U-CPME	7.7
PT-CLF	49.9
U-CLF	12.4
PT-MEK	12.8
U-MEK	12.2

^1^ The E factor was calculated assuming that the extraction solvent was completely recycled.

**Table 2:** MIC values of the various extracts obtained with different solvents against strains of *E. coli*, *B. megaterium*, and *B. subtilis*.

Extracted Sample	MIC (µg/mL)		
	*E. coli*	*B. megaterium*	*B. subtilis*
PT-DMC	118.02 ± 6.17	356.52 ± 109.46	63.91 ± 32.81
U-DMC	29.74 ± 12.57	22.02 ± 1.45	17.18 ± 2.67
PT-CPME	139.28 ± 34.35	638.87 ± 8.69	716.12 ± 1.62
U-CPME	4.89 ± 0.05	38.05 ± 9.17	43.11 ± 17.18
PT-MEK	207.37 ± 6.67	280.92 ± 27.10	29.05 ± 1.19
U-MEK	22.68 ± 2.24	4.18 ± 0.42	2.39 ± 1.05
PT-CLF	860.63 ± 1.90	750.60 ± 3.61	7490 ± 3186 ^a^
U-CLF	32.66 ± 8.51	37.58 ± 0.32	25.79 ± 0.28
VAN ^b^	1.55 ± 0.03	36.25 ± 1.09	2.63 ± 0.23

^a^ This value was assumed as a lack of antimicrobial activity. ^b^ Vancomycin (VAN) was tested as positive control.

**Table 3:** GC-MS analysis. Molecular assignment of selected peaks of the GC-MS analysis.

RT	Compound ^1^
6.00	Unknown
6.71	Unknown
9.71	Unknown
10.45	Unknown
12.29	2,6-ditert-butylcyclohexa-2,5-diene-1,4-dione
12.42	2,6-ditert-butyl-4-hydroxy-4-methylcyclohexa-2,5-dien-1-one
13.00	2,6-ditert-butyl-4-methylphenol
13.15	Unknown
13.80	Unknown
16.16	Unknown
16.78	Unknown
18.99	Unknown
19.48	Unknown
21.94	Unknown
27.48	bis(2-ethylhexyl) benzene-1,4-dicarboxylate

^1^ Molecules were attributed to the corresponding peak via NIST library identification and by imposing a mass spectrum similarity of >90%.

## Data Availability

Data are contained within the article and Supplementary Materials.

## Supplementary Materials

The following supporting information can be downloaded at: https://www.mdpi.com/article/10.3390/biotech14010022/s1, Figure S1: Post hoc Tukey test conducted on chlorophyll removal experiment; Figure S2: Post hoc Tukey test conducted on the experiment involving the preparation of extracts using green organic solvents; Figure S3: GC-MS Chromatograms of various extracts; Figure S4: Differential GC-MS chromatograms of various extracts; Table S1: Overview of the ANOVA results; Table S2: Comprehensive overview of the PCA results.
