# Supplementary material for: Identifying Antimicrobial Agents from Chlorella sorokiniana: A Biotechnological Approach Utilizing Eco-Friendly Extraction and Characterization Methods
Source: BioTech (Basel). 2025 Mar 18;14(1):22. doi: 10.3390/biotech14010022 (PMC11939975; doi:10.3390/biotech14010022)
Supplement: Supplementary file 1 [file biotech-14-00022-s001.zip › biotech-3484072-supplementary.pdf]

Supplementary Material

Identifying antimicrobial agents from *Chlorella sorokiniana*: a biotechnological approach utilizing eco-friendly extraction and characterization methods

Methods

Antimicrobial tests of the extracts (*broth dilution method*)

Minimum inhibitory concentrations were calculated using the dose–response curve (DRC) model in R. To process the data, a dose–response model was employed, which is a statistical model used to analyze response data to increasing doses of an agent or substance. The dose–response model allows for describing and estimating the relationship between the dose of an agent and the observed biological or chemical response. By using this model, it is possible to estimate the parameters of the dose–response curve through nonlinear regression analysis. This provides information on the potency and efficacy of an agent, as well as enables comparisons between different conditions or doses to evaluate any significant differences.

Statistical Analyses

Statistical analyses played a crucial role in interpreting and validating the results obtained in the present scientific study. Several statistical techniques were employed to analyze the data and derive meaningful conclusions (Diggle, 2015).

Analysis of Variance (ANOVA): ANOVA was used to compare the means of three or more groups. Specifically, a one-way ANOVA was conducted when comparing three or more independent groups on a single variable, while a two-way ANOVA was employed to analyze the effect of two factors, considering their individual effects and interaction. ANOVA allowed for the evaluation of whether there were significant differences among the different pre-treatments or experimental conditions (Cuevas et al., 2004).

Tukey and Duncan Tests: After conducting ANOVA and confirming significant differences among the groups, Tukey and Duncan tests were performed as post-hoc tests. These tests identified which specific groups differed from each other, providing a more detailed assessment of multiple differences among group means. The importance of these analyses lies in their ability to provide an accurate assessment of differences among the pre-treatments or experimental conditions tested in the study. The proper application of these statistical techniques allowed for dependable and scientifically valid conclusions. Tukey and Duncan tests are both post-hoc tests used after an analysis of variance (ANOVA) to determine which specific groups show significant differences among them.

The Tukey test, also known as Tukey's honestly significant difference (HSD) test, compares all pairs of means among the groups. This test calculates a confidence interval for the difference between the means of each pair of groups. If the difference between two means is greater than the calculated confidence interval, then the

means are considered significantly different (Keselman and Rogan, 1977). The Duncan test, also known as Duncan's multiple range test, is like the Tukey test but differs in the procedure for calculating significant differences among group means. In this test, the means are ranked by size and assigned letters (a, b, c, ...) in descending order. Groups with means that do not differ significantly are assigned the same letter, while significant differences among means are indicated by different letters (Tallarida and Murray, 1987).

In summary, the letters in the Duncan test provide a visual model to identify which groups have significantly different means from each other, while the Tukey test calculates confidence intervals to determine significant differences among all pairs of groups. Both tests are widely used for the comparison of multiple means after an analysis of variance (ANOVA).

Table S1. Overview of the ANOVA results from the chlorophyll removal, extraction yield, and antimicrobial activity (MIC) analyses, offering valuable insights into the significance of the observed differences among the experimental groups.

Experiment	Way	Groups	Df	Sum Sq	Mean Sq	F value	Pr(>F) [a]
**Chl removal**	One	Solvent	3	3908	1302.6	24.62	0.000216 ***
		Residuals	8	423	52.9		
**Extraction yields**	Two	Solvent	3	8572	2857	5.101	0.011479 *
		Pre-treatment	1	420	420	0.750	0.399150
		Solvent–Pre-treatment	3	21221	7074	12.628	0.000173 ***
		Residuals	16	8963	560		
**MIC**	Three	Bacteria	2	5301153	2650576	3.418	0.040023 *
		Solvent	4	8863909	2215977	2.858	0.032046 *
		Pre-treatment	1	7406842	7406842	9.551	0.003157 **
		Bacteria–Solvent	8	21522294	2690287	3.469	0.002696 **
		Bacteria–Pre-treatment	2	6237369	3118685	4.022	0.023544 *
		Solvent: Pre-treatment	3	7678832	2559611	3.301	0.027063 *
		Bacteria–Solvent–Treatment	6	20818814	3469802	4.474	0.000957 ***
		Residuals	54	41876130	775484		

[a] Signif. codes: 0 "***", 0.001 "**", 0.01 "*", 0.05 ".", 0.1 "", 1.

Table S2. Comprehensive overview of the PCA results.

**Principal Component Analysis**	**PC1**	**PC2**
**Eigenvalues**	0.00026	0.00006
**Total variance (%)**	62	19
**Cumulative eigenvalues**	0.00020	0.00027
**Cumulative variance (%)**	61	81
**Matrix of transformed data**		
Variable	**PC1**	**PC2**
**PT-DMC**	-0.003	-0.014
**PT-CPME**	0.032	0.0007
**PT-CLF**	-0.002	-0.005
**PT-MEK**	-0.007	0.003
**U-DMC**	-0.007	0.002
**U-CPME**	-0.008	0.002
**U-MEK**	-0.001	0.011

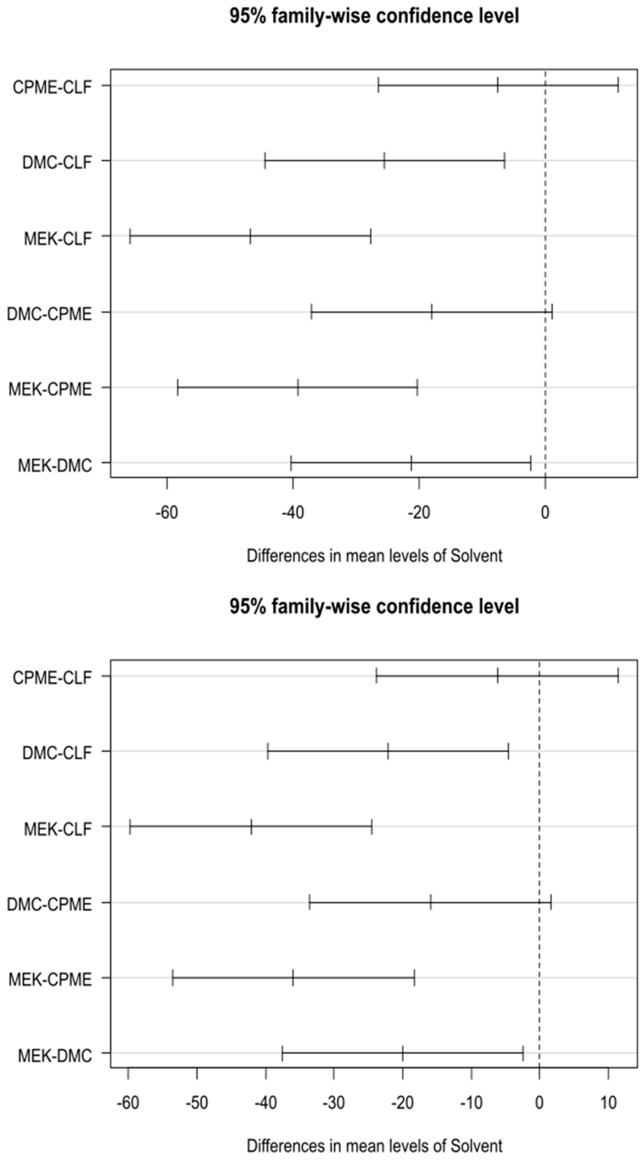

Figure S1. Post-hoc Tukey test conducted following one-way ANOVA analysis on the Chl-a (top side) and Chl-b (bottom side) removal experiment. The plot visualizes pairwise comparisons of mean values among multiple groups, revealing significant differences ($p < 0.05$). The analysis was performed to assess variations in solvent extractions among the experimental conditions.

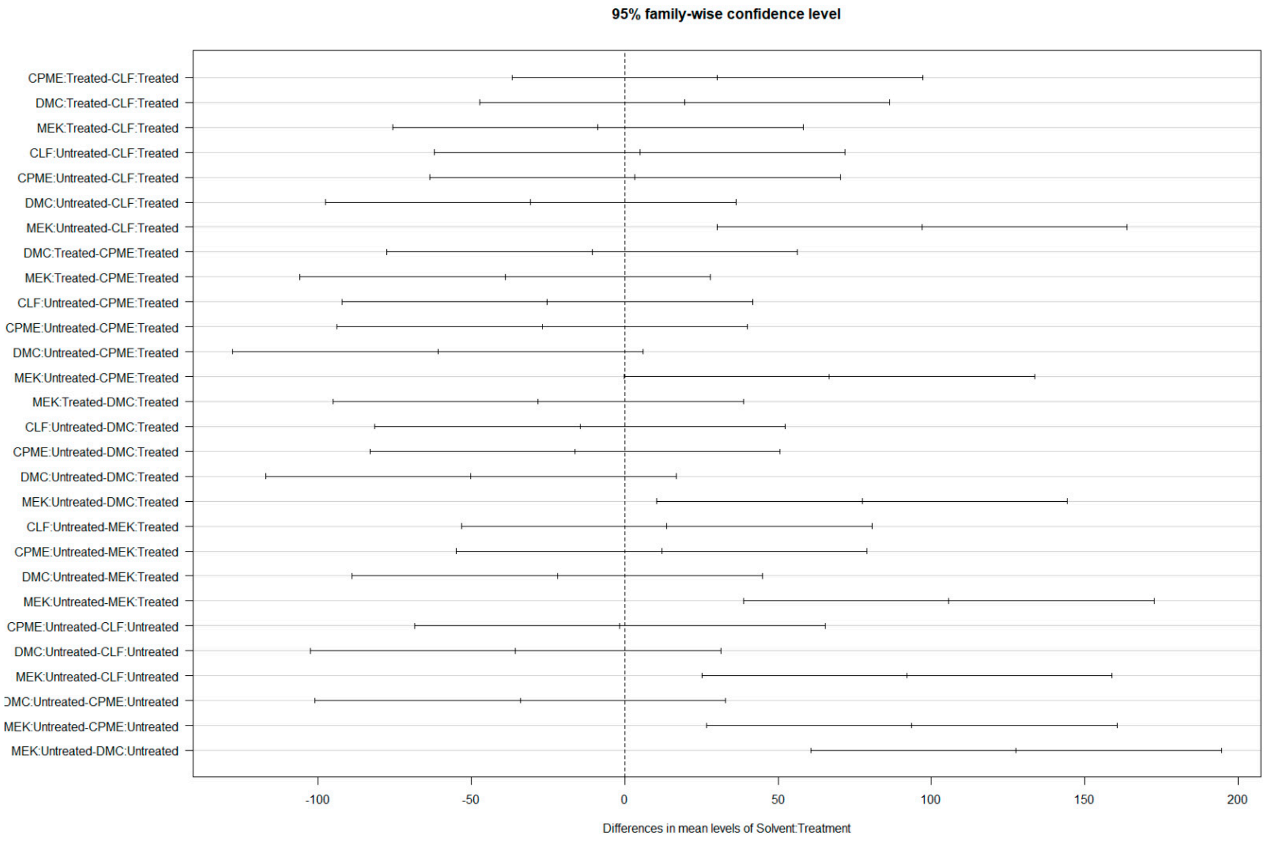

Figure S2. Post-hoc Tukey test conducted following two-way ANOVA analysis on the experiment involving the preparation of extracts using green organic solvents. The plot visualizes pairwise comparisons of mean values among multiple groups, revealing significant differences ($p > 0.05$). The analysis was performed to assess variations in solvent–pre-treatment in extract preparation among the experimental conditions.

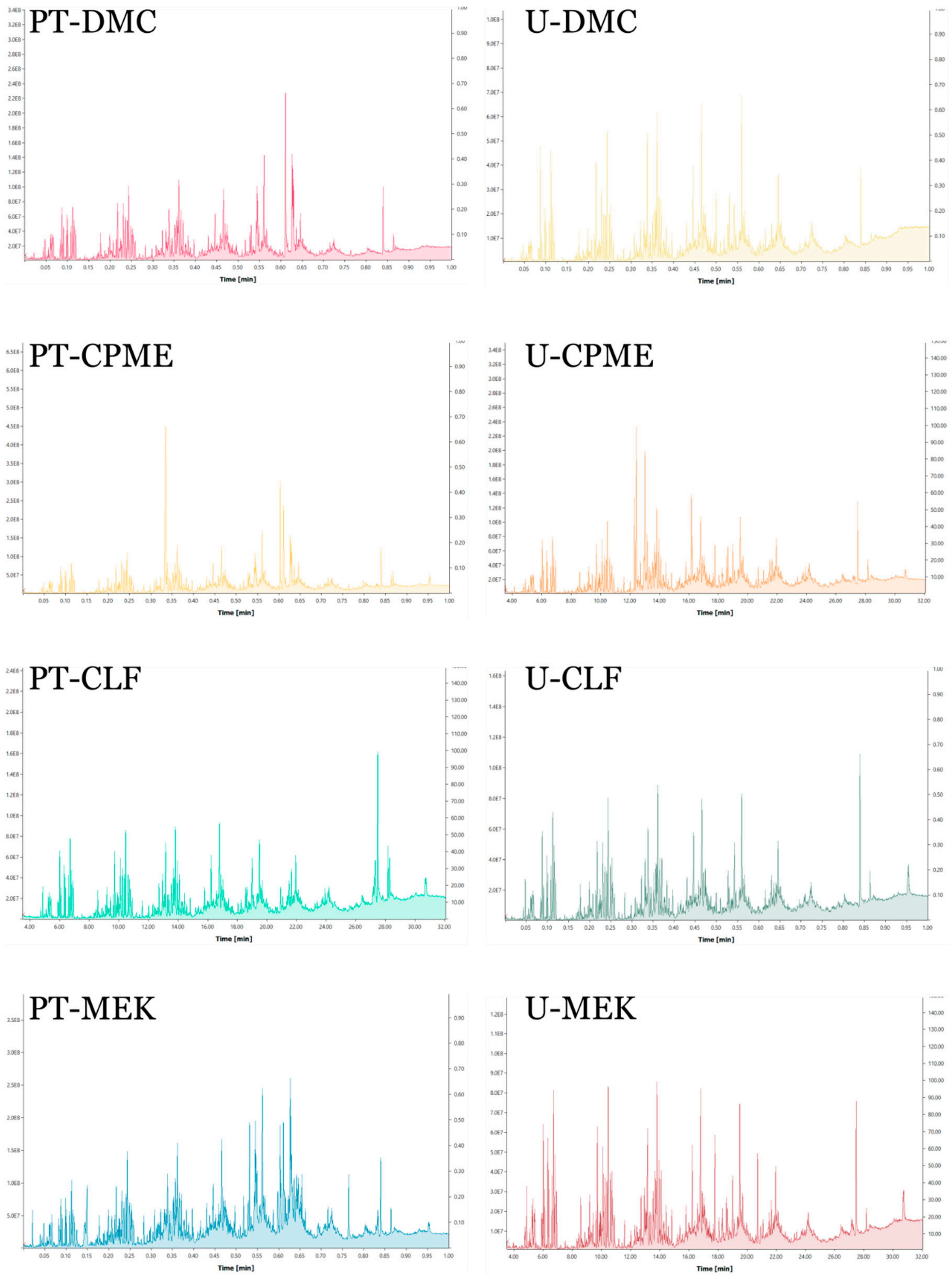

Figure S3. GC-MS chromatograms of various extracts recorded from 3 minutes up to 33 minutes. The analyses were initiated from three minutes to ensure accurate visualization of volatile molecules present in the samples. This initial time interval is crucial for capturing lighter and volatile substances, allowing for a comprehensive and sensitive assessment of compounds present in the studied samples. Recording chromatograms up to 33

minutes enables the examination of both early and late compounds, thereby providing a complete overview of the chemical composition of the analyzed extracts.

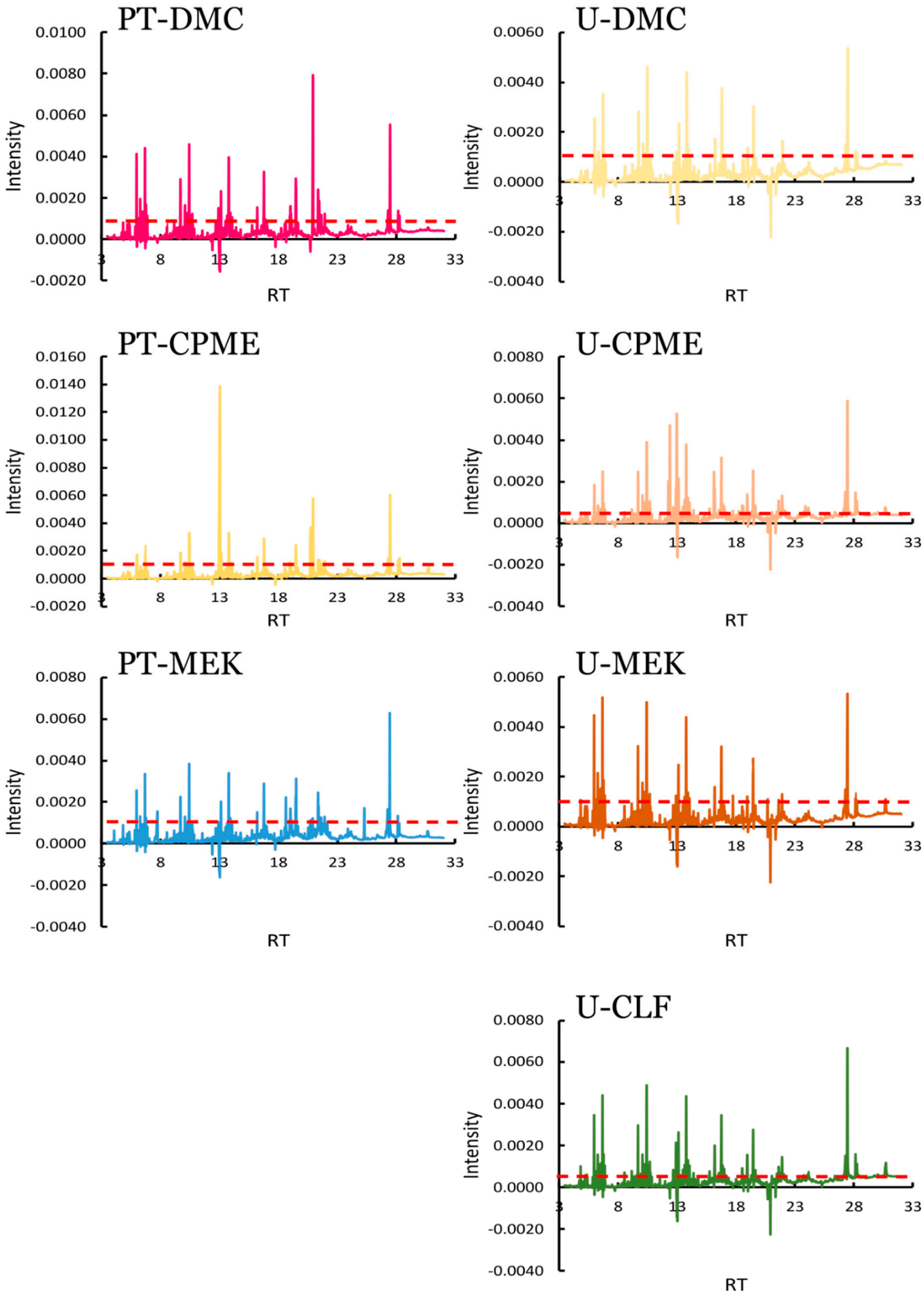

Figure S4. Differential GC-MS chromatograms of various extracts obtained subtracting the PT-CLF chromatogram from the specified extract chromatogram. These differential chromatograms were obtained to identify molecules potentially responsible for antimicrobial activity in microalgae extracts. After the normalization and calculation of mean peak intensities obtained from GC-MS analysis, it was observed that the PT-CLF extract did not exhibit antimicrobial activity. To highlight molecules present in other extracts or in higher quantities, a subtraction method was employed. The red lines represent the pre-treated extracts minus the mean, while the mean indicates the untreated extracts. This method facilitated the identification of molecules potentially responsible for the antimicrobial activity of the extracts, providing valuable insights into the chemical composition and antimicrobial activity of the analyzed samples.
